# Digital health in humanitarian crises: A case study of Gaza and the West Bank

**DOI:** 10.1177/20552076251365010

**Published:** 2025-08-21

**Authors:** Shayan Ali, Bilal Irfan, Wesam Abdeljaber, Elias Nasser, Muaaz Wajahath, Mosab Nasser, Khaled J Saleh

**Affiliations:** 1 7323 FAJR Scientific, Houston, TX, USA; 2 Austin College, Sherman, TX, USA; 3 North Texas Medical Research Institute, Rockwall, TX, USA; 4 Center for Bioethics, Harvard Medical School, Boston, MA, USA; 5 Center for Surgery and Public Health, 1861 Brigham and Women's Hospital, Boston, MA, USA; 6 Department of Neurology, 12266 University of Michigan Medical School, Ann Arbor, MI, USA; 7 Texas Tech University Health Science Center El Paso, El Paso, TX, USA; 8 The University of Texas Southwestern Medical Center, Dallas, TX, USA; 9 12268 Michigan State University College of Human Medicine, East Lansing, MI, USA; 10 2954 Wayne State University, Detroit, MI, USA; 11 5649 Central Michigan University, Mount Pleasant, MI, USA

**Keywords:** Digital health technology, electronic medical records, Gaza, West Bank, conflict zones

## Abstract

Digital health technology (DHT) has become an important aspect of healthcare systems due to its ability to improve patient outcomes and access to patient data, thereby increasing provider efficiency. However, in areas of armed conflict such as the occupied Palestinian Territories (oPT), the implementation of sustainable DHT is difficult due to Israeli military policies, which have resulted in weakened healthcare infrastructure, intermittent electricity, and restrictions on the freedom of movement. This study investigates the impact of DHT on healthcare delivery in the oPT with an emphasis on electronic medical records (EMRs), communication methods, and logistical challenges. Our findings showcase that in the West Bank, restricted EMR access and training for visiting healthcare workers (HCWs) resulted in medical students serving as scribes, while EMR crashes caused delays in healthcare delivery. In Gaza, the devastated healthcare infrastructure has resulted in the use of paper records and cell phone photos to store patient data. Language barriers and intermittent power outages compounded the difficulty of providing care. WhatsApp was a major platform for clinician contact in both locations; however, substandard cell phone connections caused communications deficiencies. To increase DHT integration, this study suggests creating an offline EMR system, a patient database, and an enhanced communication system that utilizes satellite Wi-Fi. There are benefits to incorporating an English-to-Arabic medical terminology guide within the EMR system to aid in overcoming the language barrier. Strengthening DHT in Gaza and the West Bank is crucial for improving patient outcomes, ensuring data accessibility, and enabling future research. Collaboration between international non-governmental organizations, the Palestinian Ministry of Health, and international health organizations is critical for reconstructing the healthcare infrastructure and improving health systems in Palestine.

## Introduction

Digital health technology (DHT) has become a crucial aspect of healthcare infrastructure in the past two decades, yielding improved patient outcomes, providing convenient access to test results, and enhancing patient safety.^1^ DHT includes, but is not limited to, electronic medical records (EMRs), telemedicine, and communication platforms. These tools grant healthcare providers the ability to collect patient data, utilize remote communication to support patients’ health, and allow for instant communication and data sharing amongst providers.^2–4^ EMRs are an important subset of DHT, in that they play a pivotal role in improving the quality of care and enhancing efficiency.^5^ Before the development of EMR, patient encounters were documented largely on paper and stored away in massive networks of files and physical storage. This use of paper records has substantially declined in high-income nations (HICs) with the advent of the standardization of EMR.^6,7^

In many low- and middle-income countries (LMICs), especially areas of armed conflict, such as the occupied Palestinian Territories (oPT), including Gaza and the West Bank, paper records are still used extensively due to weakened or damaged healthcare infrastructure, which hinders the effective use and implementation of EMR.^8^ As a result, the quality of care can be undermined, given that paper records are susceptible to loss, damage, deficient data security, insufficient data backup, and errors in data representation.^9,10^ Reliable data is essential for healthcare providers and international medical organizations to accurately assess injury mechanisms, mortality rates, and morbidity, thus allowing them to formulate targeted humanitarian responses.^11–14^ Although partially functional EMRs are in place, the lack of standardization and limited accessibility across hospitals hinder providers’ ability to navigate these systems effectively. This challenge is further compounded by intermittent power outages, inconsistent cellular network, and a shortage of healthcare workers (HCWs), all of which severely hinder the implementation of DHT.^15–17^ This makes it increasingly difficult for providers to access patient records and provide well-informed care. DHT has the potential to drastically improve healthcare outcomes if the challenges present in armed conflict zones can be mitigated to some degree, such as transitioning to offline systems, improved training of HCWs on operating such systems under extreme constraints, and satellite-based communications networks; these would in turn enable providers to track patient diagnostics, outcomes, imaging, and lab results in a manner that could substantially improve individual patient and population health.

Digitally accessible patient data is important for experts to conduct studies on under-researched regions like the Middle East and North Africa (MENA).^18^ This study examines how healthcare providers used DHT during medical missions in Palestine amid ongoing armed conflict, specifically focusing on the challenges encountered by the non-governmental organization (NGO) FAJR Scientific in Gaza and the West Bank from April through October 2024.

## Methods

This study was a qualitative case study utilizing observational data and testimonies of 20 HCWs who participated in one or more of FAJR Scientific's medical missions to Gaza and the West Bank from April to October 2024. HCWs included physicians, nurses, physician assistants, and medical students. All HCWs whose testimonies were considered had been to Gaza or the West Bank during the course of the Gaza war (2023-ongoing). Data were collected from semi-structured HCW interviews elucidating their field observations from the mission. The data was then analyzed to determine thematic similarities and differences for DHT implementation between the two regions. Participant ages, roles, and experiences are outlined in Table 1.

**Table 1.** Anonymized participant characteristics by mission region.

Participant ID	Role	Age at time of mission	Years of clinical experience	Mission region
A	Hip & Knee Replacement Surgeon	40	9	West Bank
B	Urologist	49	21	West Bank
C	Plastic Surgeon	55	28	West Bank
D	Internal Medicine Physician	32	6	West Bank
E	Orthopedic Adult Reconstruction Surgeon	63	23	West Bank
F	CRNA (Anesthesia Provider)	33	3	West Bank
G	RN First Assist/Director of Nursing	56	35	West Bank
H	Anesthesiologist/Pain Medicine	38	14	West Bank
I	Orthopedic Surgeon	41	17	West Bank
J	Pediatrician	57	30	West Bank
K	Orthopedic Surgeon/Co-Team Lead	57	20	West Bank
L	Orthopedic Resident	31	3	West Bank
M	Anesthesiologist/Critical Care Medicine	41	12	Gaza
N	Anesthesiologist/Intensivist	43	18	Gaza
O	Internal Medicine/ ICU/ER	48	24	Gaza
P	Anesthesiologist	49	24	Gaza
Q	Orthopedic Surgeon	48	23	Gaza
R	Anesthesiologist	53	21	Gaza
S	Orthopedic Resident	29	4	Gaza
T	Anesthesiologist	38	15	Gaza

### Data collection

Semi-structured interviews with open-ended questions were conducted with 20 HCWs who had served in one or more hospitals across Gaza and the West Bank. The objective of the interview was to understand the challenges encountered and solutions proposed by the HCWs. The interview was based on field observations by clinicians in the operating room, inpatient wards, outpatient clinics, and emergency departments, and general themes from their mission work. The interview consisted of eight open-ended questions, and was conducted via phone or Whatsapp. Phone interviews lasted an average of 20–30 minutes. Text-based responses were formatted and analyzed alongside phone transcripts. All participants gave informed consent for their responses to be used in this analysis.

### Data analysis

All interviews were transcribed verbatim initially and were later analyzed for thematic content to aid in interpreting data. Two authors worked independently to code the transcripts. The analysis was inductive; however, some deductive coding was used as well. We followed the steps outlined by Braun and Clarke, which included in-depth and rigorous thematic analysis of the transcripts, developing themes, and refining them for clarity and consistency. This allowed us to capture the insights we obtained from the participants, but also how they experienced the presence or absence of DHT in conflict zones. Interviewers weren’t involved in patient care in order to minimize bias in responses.

## Logistical challenges with EMR use and training

During medical missions to Palestine, one of the biggest obstacles identified was limited training for international and Palestinian physicians with usage of a new EMR system, as well as difficulty in accessing the EMR from hospital Wi-Fi. FAJR Scientific aimed at improving documentation protocols by developing a new EMR to be utilized by medical staff at various hospitals during the course of their missions. However, certain Palestinian hospitals lacked the capacity to implement an EMR system due to staff shortages, training and system misalignment, and internet disruptions. For example, providers in the United States typically undergo a full day of in-service EMR training when joining a new hospital; the short duration of some medical missions (less than two weeks) made this infeasible by cutting into limited time to address the residents’ healthcare needs.

Recent scholarship on short-term medical missions warns that “bolt-on” digital tools can do more harm than good when they are layered onto, rather than woven into, local information systems. A review of 152 studies found that fewer than half described how visiting teams linked their activities to the recipient health-care system, indicating that many missions overlooked potential disruption or duplication of local services.^19^ There is limited accountability, weak follow-up mechanisms, and scant evidence of a lasting benefit to the overall health system (distinct from the potential benefits of short-term capture of healthcare needs) in international medical missions “parachuting” in electronic record platforms without parallel investments in local training or governance.^20^

### Challenges with EMR in the West Bank

Due to the EMR's inability to act as an effective complement and translation to local healthcare records and EMR systems, international clinicians partnered with Palestinian medical students and residents to act as scribes, interpreters for patient encounters, and supporters in retrieving patient records. The implementation of medical students as scribes, while effective, can lead to subtle documentation errors, as a specialist in the field will have more insight into the patient's condition in comparison to a medical student.^21^ A provider on the trip stated, “I would document patient encounters on paper, then hand them off to the local medical students to enter into the EMR later” (Physician A, West Bank, 2024). Such notes were entered into existing hospital records and later added to EMR by members of the international medical team. Another persistent issue was the EMR crashing when providers were loading patient charts, delaying patient care, and increasing wait times. A physician shared, “One of the biggest challenges was not being able to dictate patient encounters as it really slowed things down” (Physician B, West Bank, 2024). This left him reliant on local medical students for transcription. He reported, “I could have seen significantly more patients if a dictation feature had been available” (Physician B, West Bank, 2024). These experiences showcase how minimal EMR training hinders providing effective patient care in conflict zones.

### Challenges with EMR in Gaza

The impedance of DHT in Gaza is influenced by the continuous Israeli assault on hospitals, inflicting widespread damage to the healthcare infrastructure.^22,23^ This has led some Palestinian HCWs to initiate an archive of patient records on notebooks. Due to the high volume of incoming trauma, an international provider stated that “there wasn’t always time or resources to document patient encounters” (Physician C, Gaza, 2024). Due to the absence of a functioning EMR, providers were unable to centrally access patient imaging, which at times may have been conducted at a different hospital from which they were now displaced, or was inoperational.^24^ As a result, patients often presented their imaging, or screenshots of it, to providers on their smartphones. However, this approach was constrained in itself due to loss of phones, difficulty in sending images to a provider via text due to lack of WiFi, or the lack of battery charge (and available options to charge a phone) to be able to access such images.^25–27^ This method of paper medical records showcases how damaged healthcare infrastructure drastically undermines healthcare standards in conflict zones. The dilemma in developing and maintaining a competent EMR system is underfunding exacerbated by Israeli restrictions on aid, relentless attacks on hospitals, and the utilization of outdated technology.^28,29^ Additionally, another obstacle in Gaza is the continuous targeted killing of HCWs, causing a dire shortage of specialists and HCWs who would otherwise aid in the development and implementation of DHT. Take, for example, the killing of Dr Saeed Joudeh, who was the last orthopedic surgeon in North Gaza, and would have been poised to assist international medical teams in such efforts.^30^ The lack of an orthopedic surgeon poses a grave threat to the healthcare system, as a substantial number of patients suffer from musculoskeletal injuries.^31^ Thus, the Palestinian population in Gaza is ever more reliant upon medical relief organizations and humanitarian actors to bring international orthopedic surgeons to treat patients, thereby necessitating a further need to document such cases.

### Communication methods and language barriers

Communication is one of the most fundamental aspects of delivering adequate healthcare; this is supported by WhatsApp, despite its weaknesses in end-to-end encryption, in Palestine, and facilitates communication between providers and HCWs regarding patient care.^32^ A provider mentioned that “patient data would also be shared amongst providers in an encrypted file on WhatsApp” (Physician D, Gaza, 2024). However, this would be rendered useless in the absence of cellular reception. In addition to this, language barriers presented a challenge in patient interactions. Palestinians natively speak Arabic, often resulting in misunderstandings in the absence of an interpreter. To combat this issue, multiple providers shared that they “relied on local medical students and residents for interpretation” (Physician E, Gaza, 2024). To overcome this hurdle, providers often relied on Google Translate, which helped streamline patient care.^33^

Because of the limited infrastructure in Gaza, WhatsApp was the primary method of communication in Gaza and was used for communication amongst team members and hospital staff. This builds upon an existing practice among Palestinian healthcare workers in using WhatsApp for day-to-day clinical encounters. Some practitioners had made specialty-specific WhatsApp groups to share updates on patients and help establish some form of continuity of care for the incoming medical teams. However, at times, power outages or limited cellular reception made the app inaccessible.

## Recommendations for improving digital health integration

### West Bank

The fundamental difference between the Gaza and West Bank missions was that Gaza was focused on trauma cases, whereas the West Bank was largely concentrated on elective surgeries. Prior to the West Bank mission, international providers were notified of the types of cases they would be performing. To give physicians a better understanding of the patients they will be performing surgeries on, Palestinian physicians shared records in a secure manner so practitioners thoroughly understand the patients they will be operating on. This is an important step and aided in streamlining services at hospitals where this pre-mission contact was successfully conducted. Additionally, a centralized patient database should be developed to improve surgical planning. To provide a better understanding and context of patients, cases can be presented to international physicians over Zoom meetings, which was shown to be a viable option during the pandemic in Thailand.^34^ To address the sub-optimal EMR in the West Bank, a comprehensive system needs to be developed with the ability to upload patient imaging, lab results, and establish some form of inter-connectivity between the hospitals to improve patient care, as checkpoints restrict the lives of Palestinians and can rapidly change which hospital or healthcare service is accessible to them.^35^ To resolve the challenges present with a language barrier, international physicians should be given a short manual on medical terminology, which should also be readily available on the EMR. Furthermore, physicians should have the convenience of dictating patient encounters. To overcome the challenges presented by the EMR crashing, subsequent EMRs should have an offline feature enabling providers to upload patient information and retrieve patients' charts. This has shown to enhance continuity of care and improved efficiency in low-resource settings.^36^ To ensure consistent communication, providers should be educated on cellular plans that will work in the West Bank. This will enhance communication and assist in patient coordination and care. Future medical missions should also explore using satellite Wi-Fi as it allows for stable reception regardless of location, power outages, and can be implemented in conflict-affected zones where there is poor infrastructure.^37,38^ More importantly, the EMR systems should be user-friendly so providers do not need to spend valuable time learning the EMR system, thus providers will be more focused on supporting the healthcare needs and systems in the West Bank.

### Gaza

HCWs visiting Gaza faced profound challenges that drastically impacted the healthcare infrastructure and local population. To address the major healthcare deficiency, which is the lack of an EMR system, NGOs and international healthcare stakeholders like the World Health Organization (WHO) may collaborate with Palestinian hospitals in developing an EMR system that has offline functionality, making it operable in the event of a power outage. The system needs to be user-friendly so visiting physicians can operate it effectively. Additionally, to address the language barrier, the EMR should include a short English-to-Arabic medical terminology guide to ensure communication with patients. Collaborations with the Palestinian Ministry of Health to develop programs for medical and nursing students to become professional medical interpreters may also be considered. This is important as trained interpreters can help improve patient outcomes and satisfaction, in contrast to untrained ones.^39^ Additionally, cellular connection is a concern for communication amongst the HCW. To overcome this obstacle, providers should carry small communication devices like pagers or walkie-talkies. This strategy can be easily implemented, as pagers were a common method of communication in hospitals until smartphones took over. In addition to this, a secure communication system can be integrated into the EMR system; this was successfully implemented in the UK and led to increased efficiency.^40^ Pagers and EMR-based communication could be used in tandem with one another, with the EMR communication being used for non-critical situations, and the pagers being used in the event of an emergency. By combining offline EMR functionality and communication backup methods, HCWs in Gaza may be able to better manage the influx of trauma cases.

### Strategies forward

Implementing DHTs in Gaza and the West Bank is important to improving patient outcomes and mapping the healthcare needs of the populace. The testimonies of international HCWs who have served in these areas present the need for accurate data collection, stable communication networks, competent EMR systems with offline capabilities, and interpreters to address the language barrier. Collaborations between the Palestinian Ministry of Health, NGOs, and international aid agencies may offer avenues for uplifting and reconstructing the digital healthcare infrastructure in Palestine.

Importantly, however, guidance for digital health implementation framed for high-income-country actors therefore needs to consider and be anchored in long-term, co-created partnerships that elevate Palestinian clinicians as equal co-designers, by explicitly cautioning against “surgical colonialism” and parallel data systems that sideline national priorities.^41^ Digital-health governance requires a stressed focus to be placed on ethical oversight, shared data stewardship, and context-specific consent procedures to be in place from the outset to protect vulnerable populations and prevent exploitation of conflict-affected groups; these safeguards can only be achieved through genuine stakeholder engagement within Gaza and the West Bank.^42^ Some recommendations are outlined in Table 2.

**Table 2.** Recommendations for digital health technology implementation in Palestine.

Category	Recommendations	Region(s)
EMR systems	Develop offline-capable EMRs that can operate without continuous internet or electricity	West Bank and Gaza
	Integrate functionalities for uploading diagnostic imaging (x-ray, MRI, and ultrasound) and lab results	
	Create a centralized, interoperable EMR accessible across multiple hospitals	
	Ensure EMR platforms are user-friendly to minimize training time for providers	
	Incorporate an integrated English-Arabic medical terminology glossary	
	Enable a dictation feature to streamline and expedite patient documentation	
Communication	Implement satellite-based Wi-Fi to ensure consistent connectivity, independent of local infrastructure	West Bank and Gaza
	Establish specialty-specific WhatsApp groups for enhanced care coordination and continuity	
	Introduce secure EMR-integrated messaging platforms for efficient and secure communication	
	Adopt pager or walkie-talkie systems for emergency communications backup	Gaza
	Conduct pre-mission virtual meetings via Zoom or similar platforms to enhance provider preparation	West Bank
Language barriers	Provide professional medical interpretation training programs in collaboration with local educational institutions	West Bank and Gaza
	Integrate automated translation tools within EMR systems	
Coordination and preparation	Securely share detailed patient records and case histories with international teams prior to medical missions	West Bank
	Foster long-term collaborative partnerships involving local stakeholders and international NGOs for system development	West Bank and Gaza
	Train local healthcare workers extensively on newly introduced digital health technologies	
Infrastructure	Collaborate with NGOs and the WHO to systematically rebuild digital health infrastructure	Gaza
	Provide portable power solutions (solar-powered or battery-operated systems) for maintaining EMR and communication tools	
Data governance	Establish transparent, context-specific consent processes for patient data handling	West Bank and Gaza
	Implement robust data stewardship frameworks emphasizing Palestinian ownership and ethical oversight	
	Regularly engage Palestinian stakeholders to ensure alignment with local health priorities	

EMRs: electronic medical records; MRI: magnetic resonance imaging; NGO: non-governmental organization; WHO: World Health Organization.

## Discussion

The findings of this study build upon prior research conducted on digital health in conflict zones. For example, during the Syrian Civil War, international NGOs implemented various communication platforms, notably WhatsApp, to enhance healthcare services in the war-torn region.^43^ The Yemeni civil war similarly presented the necessity for competent communication and digital health systems to be implemented for adequate healthcare coverage.^44^ However, unlike many other areas affected by armed conflict, Gaza and the West Bank face compounding challenges, including the lack of digital sovereignty, Israeli control over telecommunications infrastructure, and an ever-tightening siege. In addition to this, offline EMR systems have shown promise in regions lacking power and internet, and those dealing with an acute refugee crisis.^36^ Our findings highlight that this is paramount in Gaza and the West Bank due to intermittent power outages and a lack of reliable internet connection. However, unlike other conflict zones where offline EMRs were scaled across facilities, EMR systems in the oPT often lack interoperability across hospitals, undermining continuity of care.

Nevertheless, Palestine poses unique challenges given the punitive blockade and military occupation that increasingly restricts the movement of people and resources necessary for implementing these changes. The lack of inter-hospital connectivity, and the targeted destruction of healthcare infrastructure, further complicates care coordination and serves to fragment healthcare in an already deprived region. Certain lessons have been learned from the mission, as summarized below.

**Table:** 

Lessons learned for digital health technology implementation in conflict zones
• WhatsApp served as a communication platform, despite its weaknesses in encryption, which in turn helped coordinate care in regions where healthcare infrastructure was deficient and connectivity was an issue.
• Overreliance on medical students and residents for EMR transcription and patient encounter interpretation can pose risks of clinical inaccuracy.
• Incorporating dictation tools into EMR can improve provider efficiency, thereby increasing provider consultations.
• Satellite Wi-Fi and offline-capable systems are important in conflict zones for the continuity of care.
• Training programs in medical interpretation and EMR orientation can mitigate operational challenges during short-term missions.
• Inter-connectivity of EMR across hospitals is essential for ensuring continuity of care, especially in fragmented healthcare systems.
• Integration of field-based feedback into DHT system design enhances relevance and effectiveness in humanitarian contexts.

## Conclusion

Future digital health interventions must be conceived as long-term, federated infrastructures that privilege offline EMR capability, satellite-backed connectivity, and tiered interpreter support while embedding robust data-governance safeguards from the outset. High-income country actors and NGOs should resist the allure of short-cycle deployments and instead invest in capacity-building pipelines that cultivate Palestinian expertise and codify shared data-stewardship frameworks. Purposive sampling allowed the inclusion of experienced clinicians with direct mission involvement, strengthening the study's relevance to digital health implementation in conflict zones. Scaling these insights will require mixed-methods evaluations that track clinical, operational, and equity outcomes over time, yet the essential lesson already stands: digital tools will improve health in crises only when they are interwoven with locally governed systems, resilient communications infrastructure, and a commitment to uphold patients’ rights even amid conflict.

Beyond clinical limitations, our findings showcase the consequences of occupation and infrastructural instability on the Palestinian healthcare system. Unchecked attacks on Gaza's hospitals have wiped out entire electronic archives, rupturing the digital backbone on which cancer registries, trauma surveillance, and any future Palestinian public-health system depend. Re-establishing a Palestinian-governed, interoperable health-information network is therefore more than a technical upgrade: it is a humanitarian imperative that underwrites the population's right to health and the credibility of global relief efforts. A case in point is that the fragmentation of DHT not only compromises care but also results in and perpetuates substandard care. Addressing lapses in DHT in the oPT must be recognized as both a medical and moral obligation.
